# Draft Genome Sequence of *Klebsiella pneumoniae* subsp. *pneumoniae* ATCC 9621

**DOI:** 10.1128/genomeA.01718-16

**Published:** 2017-03-23

**Authors:** Anja Poehlein, Hristo Najdenski, Diliana D. Simeonova

**Affiliations:** a Genomic and Applied Microbiology and Göttingen Genomics Laboratory, Georg-August University Göttingen, Göttingen, Germany; b Laboratory of Zoonoses and Bacterial Virulence, Department of Infectious Microbiology, Institute of Microbiology, Bulgarian Academy of Sciences, Sofia, Bulgaria

## Abstract

We present here the 5.561-Mbp assembled draft genome sequence of *Klebsiella pneumoniae *subsp.* pneumoniae* ATCC 9621, a phosphite- and organophosphonate-assimilating Gammaproteobacterium. The genome harbors 5,179 predicted protein-coding genes.

## GENOME ANNOUNCEMENT

*Klebsiella* spp. occur in soil, water, and on plants, and some strains are considered a part of the normal flora of the human gastrointestinal tract. However, *K. pneumoniae*, *K. oxytoca*, and *K. granulomatis* are known human pathogens causing pneumonia, nosocomial infections, bacteremia, and sepsis in patients with weak immune systems. Furthermore, *K. pneumoniae* has emerged as a severe community-acquired infectious agent, causing diseases, including pyogenic liver abscess and meningitis (1–3).

*K. pneumoniae *subsp.* pneumoniae* strain ATCC 9621 was a subject of genetic and biochemical studies related to the degradation of organophosphonates and inorganic phosphite (Pt) (4, 5).

The strain assimilates Pt as a single P source via a phosphonate dehydrogenase (PtxD)-independent pathway (4, 5) and was chosen for sequencing as a control strain in a study related to the diversity of phosphite-assimilating bacteria.

Total DNA of *K. pneumoniae *subsp.* pneumoniae* ATCC 9621 was isolated with the MasterPure complete DNA purification kit (Epicentre, Madison, WI, USA). The extracted DNA was used to generate Illumina shotgun paired-end sequencing libraries, which were sequenced with a MiSeq instrument and the MiSeq reagent kit version 3, as recommended by the manufacturer (Illumina, San Diego, CA, USA). Quality filtering using Trimmomatic version 0.32 (6) resulted in 2,751,284 paired-end reads. The assembly was performed with the SPAdes genome assembler software version 3.6.2 (7). The assembly resulted in 67 contigs (>500 bp) and an average coverage of 137-fold. The assembly was validated and the read coverage determined with Qualimap version 2.1 (8). The draft genome sequence of *K. pneumoniae *subsp.* pneumoniae* ATCC 9621 consists of a chromosome (5,561,554 bp) including an IncFII-type plasmid, with an overall G+C content of 57.17%. Automatic gene prediction and identification of rRNA and tRNA genes were performed using the software tool Prokka (9). The draft genome contained 11 rRNA genes, 76 tRNA genes, 4,347 protein-coding genes with function prediction, and 832 genes coding for hypothetical proteins. *K. pneumoniae *subsp.* pneumoniae* ATCC 9621 grows aerobically on glucose and its derivatives as carbon sources, fermentatively on lactose, and as facultative anaerobe reduces nitrate as final electron acceptor.

The phosphate requirements of the strain are covered by the low-affinity inorganic phosphate uptake system (Pit; *pitA*), the high-affinity (PstABCS) system, phosphate ester uptake system proteins (PTS), and a phosphate:Na^+^ symporter, regulated by the phosphate starvation-inducible protein PhoH and the phosphate uptake regulator PhoU. Under phosphate starvation, the strain oxidizes and assimilates phosphite and organophosphonates. The genome harbors one copy of the *phoBR* genes of the Pho regulon. Phosphonate uptake proceeds via ABC-type transporters, a general *phnCDEE* transporter and a specific transporter for 2-aminoethylphosphonate (2-AEP)-*phnSTUV*. The genome encodes a complete 2-AEP degradation pathway to acetaldehyde (*phnAWX*), a C-P lyase complex for assimilation of aklylphosphonates, and Pt (*phnFGIJKLMNP*) and the alkaline phosphatase gene *phoA* (10), involved in the uptake of phosphate esters as well. Genes involved in the oxidation of phosphite and hypophosphite (*ptx* or *htx*) were missing.

### Accession number(s).

This whole-genome shotgun project has been deposited at DDBJ/EMBL/GenBank under the accession no. MSAK00000000. The version described in this paper is version MSAK01000000.
